# Incidental detection of malignancy on point‐of‐care renal ultrasound: A case series

**DOI:** 10.1002/ajum.12340

**Published:** 2023-03-15

**Authors:** Kristen Adorno, Courtney Martin, Caroline Blatcher, Stephanie Smith, Tara Cassidy‐Smith, Sarab Sodhi

**Affiliations:** ^1^ Cooper University Hospital Camden New Jersey USA

**Keywords:** flank pain, haematuria, hydronephrosis, POCUS, renal cell carcinoma, renal ultrasonography

## Abstract

Renal point‐of‐care ultrasound (POCUS) is an increasingly common initial imaging modality in the diagnostic workup of renal colic. The primary use of renal POCUS is to assess for hydronephrosis; however, other significant findings suggestive of malignancy can also be identified. We present three cases of unexpected findings identified initially on POCUS in the emergency department, which subsequently led to new diagnoses of malignancy. As renal POCUS becomes more frequently used in clinical practice, physicians must be able to recognise abnormal images that indicate possible malignancy and the need for further workup.

## Introduction

The diagnostic workup of suspected urolithiasis has historically been based on computed tomography (CT) imaging. However, recent data have demonstrated that ultrasound, including renal point‐of‐care ultrasound (POCUS) performed by an emergency physician, is a reasonable initial imaging modality and has the advantage of being faster and reducing ionising radiation exposure for patients.^1^ For this reason, renal POCUS is being performed much more frequently in the outpatient and emergency department setting for patients with flank pain and/or haematuria.

Incidental findings are common among all imaging modalities, including CT and magnetic resonance imaging (MRI). As clinicians perform renal POCUS more routinely, it is likely that they will encounter renal and bladder masses, as well as other incidental pathology. It is important that clinicians be able to recognise potentially malignant masses that are incidentally found on POCUS. We present three cases in which POCUS was used to evaluate patients presenting to the emergency department, which revealed suspicious masses that were ultimately found to be malignant.

## Case descriptions

### Case 1

A 50 year old woman with no significant past medical history presented to the emergency department with one day of acute right‐sided flank pain associated with nausea. Her presentation was concerning for ureterolithiasis due to her unilateral flank pain and urinalysis demonstrating microhaematuria. A renal POCUS was obtained by the emergency physician.

**Figure 1:**
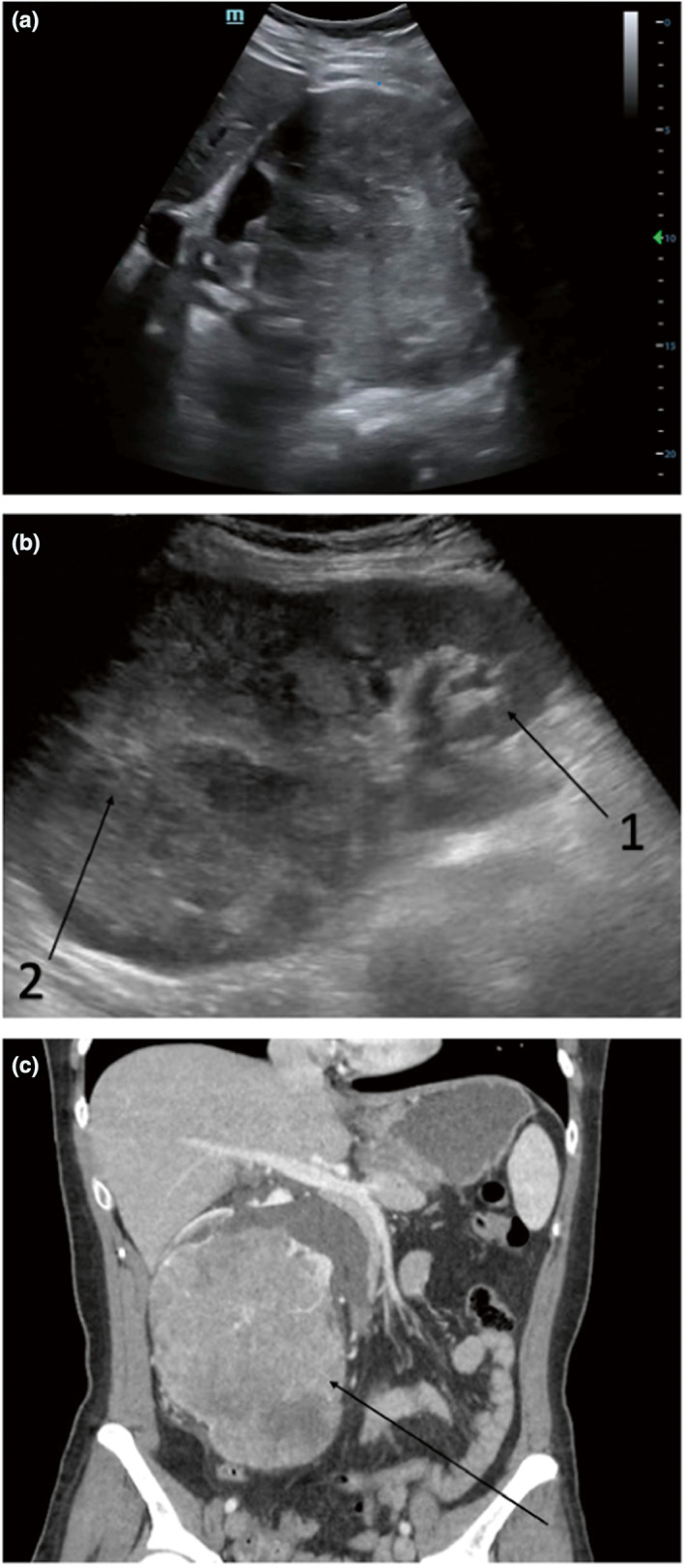
Renal cell carcinoma in the right kidney of a 50 year old woman. (a) Point‐of‐care ultrasound showing irregularity of the right renal cortex and obliteration of normal renal anatomy. (b) Radiology‐performed renal ultrasound showing a small area of remaining normal renal cortex and medulla (b1) as well as obliteration of superior renal cortex by an irregular hyperechoic mass (b2). (c) Coronal computed tomography abdomen/pelvis demonstrating right renal mass (arrow) ultimately diagnosed on pathology as renal cell carcinoma.

Sonographic examination of the right kidney revealed limited visualisation of the renal pelvis, and a large mildly hyperechoic region was noted, which appeared to be obstructing the normal renal architecture (Figure 1a). The pelvis was partially visualised and appeared slightly distended consistent with mild hydronephrosis (Figure 1b). A 12 × 12 × 12 cm hypervascular mass with mixed echogenicity was noted. This necessitated further imaging, and a CT of the abdomen and pelvis was ordered. The CT findings were concerning for renal cell carcinoma of the right kidney (Figure 1c). The patient ultimately underwent radical nephrectomy, and pathology was consistent with this diagnosis.

### Case 2

**Figure 2:**
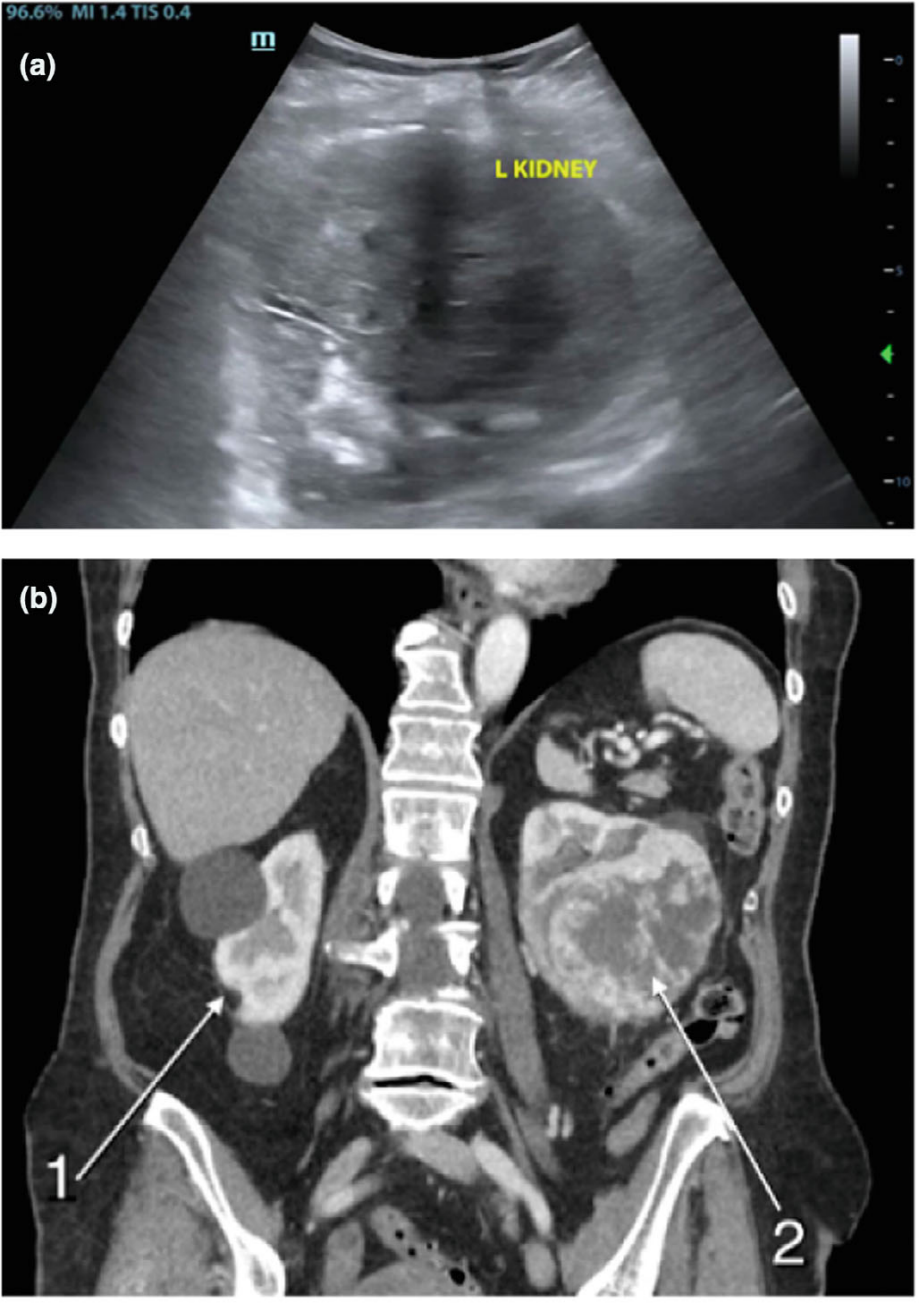
Renal cell carcinoma in the left kidney of an 83 year old woman. (a) Point‐of‐care ultrasound showing an abnormal hypoechoic region in the left renal cortex suspicious for malignancy. (b) Computed tomography scan of the abdomen and pelvis showing a normal‐appearing right kidney (1) with two renal cysts and a left renal mass (2) consistent with renal cell carcinoma.

An 83 year old woman with a history of type II diabetes mellitus, hypertension and anxiety presented to the emergency department with one day of painless haematuria and an episode of urinating blood clots, associated with urinary hesitancy. Renal POCUS was obtained, which showed a large round structure of mixed echogenicity within the left kidney appearing to obstruct the normal renal architecture (Figure 2a). A structure of mixed echogenicity was noted in the lumen of the bladder as well. A CT of the abdomen and pelvis was obtained, which showed an 8.7 cm left renal mass suspicious for renal cell carcinoma with invasion into the left renal vein and a 3 cm bladder mass and multiple liver lesions (Figure 2b). The patient was admitted to the hospital, where she underwent a biopsy that confirmed the diagnosis of renal cell carcinoma. She then underwent left radical nephrectomy.

### Case 3

**Figure 3:**
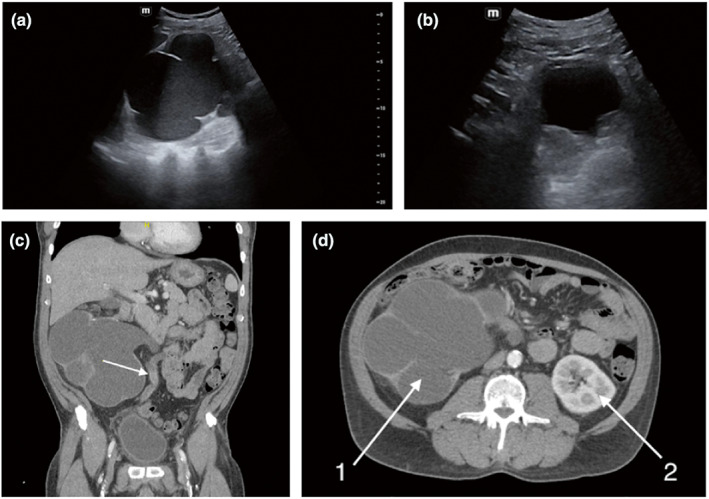
Severe hydronephrosis of the right kidney due to an obstructing bladder mass in a 62 year old man. (a) Point‐of‐care ultrasound (POCUS) demonstrating severe right hydronephrosis with near‐complete obliteration of the renal parenchyma. (b) POCUS of the pelvis showing a bladder with thickened wall. (c) Coronal computed tomography (CT) of abdomen/pelvis showing severe right hydronephrosis and significant right hydroureter (arrow). (d) Axial CT comparing right kidney with severe hydronephrosis (1) with normal left kidney (2).

A 62 year old man with a history of hypertension, hyperlipidaemia, coronary artery disease and tobacco use presented to the emergency department with 9 months of haematuria with intermittent right flank pain. Renal POCUS was obtained, which showed severe hydronephrosis of the right kidney as well as irregularity and thickening of the bladder wall (Figure 3a,b). A CT scan was obtained, which confirmed chronic severe right hydronephrosis with hyperattenuating/enhancing soft tissue in the right ureter suspicious for blood clots and/or urothelial tumour (Figure 3c,d). Computed tomography also demonstrated bladder wall thickening with perivesicular infiltration suspicious for inflammation vs. infiltrative tumour.

The patient was admitted to the hospital and underwent cystoscopy with transurethral resection of bladder tumour with partial resection due to the depth of tumour. Upon confirming a high‐grade urothelial carcinoma through pathology, the patient underwent an additional surgery for total resection, which ultimately revealed a large bladder tumour (4 cm) in the region of the bladder neck and obstructing the right ureteral orifice.

## Discussion

Point‐of‐care ultrasound plays an important role in the initial diagnostic workup of haematuria and suspected renal colic. In a multicentre, randomised, comparative effectiveness trial, Smith‐Bindman *et al*.^1^ showed that in patients with renal colic, an initial imaging modality of ultrasound was associated with lower radiation exposure and lower mean total cost without significant differences in high‐risk diagnoses, complications, pain, serious adverse events or hospitalisations. Additionally, a retrospective chart review performed by Edmonds *et al*.^2^ in 2010 showed that a normal renal POCUS is associated with a low likelihood of need for urologic intervention within 90 days for adult patients with suspected urolithiasis. In regard to the detection of hydronephrosis, Riddell *et al*. (2014)^3^ found that bedside ultrasound was 78.4% sensitive in diagnosing hydronephrosis in patients with nephrolithiasis. Due to this evidence, POCUS is becoming more commonly used by clinicians in the workup of renal colic.

When performing a POC renal ultrasound, sonographers are trained to look for hydronephrosis, which indicates a degree of urinary obstruction. However, it is possible that POCUS may additionally reveal the unexpected finding of a renal or bladder mass. We describe two cases in which POCUS was used to evaluate for hydronephrosis, which unexpectedly revealed renal masses that were subsequently diagnosed as renal cell carcinoma. In the third case, we describe a patient with severe unilateral hydronephrosis with an irregular bladder wall in the setting of prolonged haematuria. Given these sonographic findings, the severity of the hydronephrosis and the duration of the patient's symptoms, their presentation appeared more consistent with an obstructing bladder mass rather than ureterolithiasis. In patients with abnormal POCUS, differing from standard hydronephrosis, physicians should consider obtaining CT to evaluate for malignancy as early detection is beneficial in these patients.

Renal cell carcinoma represents 90% of all primary renal neoplasms.^4^ It arises from the renal cortex, whereas transitional cell carcinomas arise from the renal pelvis. Renal cell carcinoma is more prevalent in men with the median age of 64. Risk factors include smoking, hypertension, obesity, chronic kidney disease and acquired polycystic kidney disease. The five‐year survival rate is 62%.^5^ Treatment is typically surgical resection with the addition of immunotherapy or chemotherapy for advanced stages.

Ultrasound is often used by urologists to help detect renal masses. Since surgical resection is the main treatment modality, early identification of renal tumours leads to better outcomes and survival.^6^ Renal tumours can be classified as cystic, solid or complex on ultrasound and can be isoechoic, hyperechoic or hypoechoic. Right‐sided masses are more likely to be visualised on ultrasound because the liver acts as an acoustic window, and they may be discovered during ultrasound studies intended to examine the liver and gallbladder.^4^ The tumour shape and borders are often indistinct and poorly defined.

As renal POCUS becomes more frequently utilised as a first‐line imaging modality, physicians must be able to recognise abnormal images that indicate possible malignancy and the need for further workup. Any unusual finding detected on ultrasound should be noted in the medical record and be discussed with the patient, and appropriate follow‐up imaging should be pursued.

## Disclosures

This manuscript was sent to the Cooper IRB and was deemed to not require IRB review, based on their guidelines. All patients at the time of their care gave their informed consent for their information to be used in any publications that resulted, providing adequate protection of their identity was allowed for. This material is the authors' own original work, which has not been previously published elsewhere. The paper reflects the authors' own research and analysis in a truthful and complete manner.

## Authorship statement

We confirm that the manuscript has been read and approved by all named authors and that there are no other persons who satisfied the criteria for authorship but are not listed. We further confirm that the order of authors listed in the manuscript has been approved by all of us and that the authorship listing conforms to the journal's authorship policy.

## Funding

No funding information is provided.

## Conflict of interest

We wish to confirm that there are no known conflicts of interest associated with this publication, and there has been no significant financial support for this work that could have influenced its outcome. We also wish to confirm that all appropriate consent has been obtained regarding human subjects involved.
